# Using Crowdsourced Food Image Data for Assessing Restaurant Nutrition Environment: A Validation Study

**DOI:** 10.3390/nu15194287

**Published:** 2023-10-08

**Authors:** Weixuan Lyu, Nina Seok, Xiang Chen, Ran Xu

**Affiliations:** 1 Department of Geography, University of Connecticut, Storrs, CT 06269, USA; weixuan.lyu@uconn.edu (W.L.); xiang.chen@uconn.edu (X.C.); 2 Department of Allied Health Sciences, University of Connecticut, Storrs, CT 06269, USA; nina.seok@uconn.edu

**Keywords:** nutrition assessment, food image data, image recognition, crowdsourcing, validation, restaurant, food environment, Hartford, FAFH

## Abstract

Crowdsourced online food images, when combined with food image recognition technologies, have the potential to offer a cost-effective and scalable solution for the assessment of the restaurant nutrition environment. While previous research has explored this approach and validated the accuracy of food image recognition technologies, much remains unknown about the validity of crowdsourced food images as the primary data source for large-scale assessments. In this paper, we collect data from multiple sources and comprehensively examine the validity of using crowdsourced food images for assessing the restaurant nutrition environment in the Greater Hartford region. Our results indicate that while crowdsourced food images are useful in terms of the initial assessment of restaurant nutrition quality and the identification of popular food items, they are subject to selection bias on multiple levels and do not fully represent the restaurant nutrition quality or customers’ dietary behaviors. If employed, the food image data must be supplemented with alternative data sources, such as field surveys, store audits, and commercial data, to offer a more representative assessment of the restaurant nutrition environment.

## 1. Introduction

The dietary landscape of Americans has undergone a significant transformation, characterized by a growing preference for dining out over cooking at home. The last two decades have witnessed constant growth in food away from home (FAFH). In 2010, expenditures on FAFH in the United States reached 616.4 billion USD, constituting approximately 50.2 percent of the total food spending for that year. This marked a significant milestone, as the market share of FAFH surpassed that of food at home (FAH) for the first time [1]. Since 2011, FAFH has constituted more than 30 percent of consumers’ overall food energy intake [2]. The latest data from the United States Department of Agriculture (USDA) in 2022 confirms this trend, with consumers continuing to spend more on FAFH than FAH, and FAFH expenditures growing at an 8 percent annual rate [3]. This trend of diminishing home-cooked meals and a growing reliance on FAFH is expected to continue in the foreseeable future [1]. Age and income are key factors influencing individual FAFH frequency. Younger individuals, especially those aged 35–44, tend to consume FAFH more often [1]. Additionally, higher-income households both spend more on and obtain FAFH more frequently compared to lower-income households [1]. This transition in dietary behavior within specific demographics raises health concerns, as FAFH tends to be calorie-dense and lacks nutrients when compared to FAH [4]. This discrepancy arises from FAFH’s high level of total fat, saturated fat, sodium, and cholesterol levels, coupled with a lack of dietary fiber [5]. Furthermore, the consumption of FAFH has been associated with poorer diet quality and a reduced intake of essential food groups, including fruits, vegetables, and grains [6,7,8]. These imbalanced dietary patterns have been identified as significant contributors to the risk of obesity, type 2 diabetes, and cardiovascular disorders across all age groups [9,10,11,12].

To comprehensively understand the nutrition quality of FAFH and its impact on the community, it is crucial to employ appropriate methods and data for evaluating the nutritional content of the food served and the dietary choices of the customers at restaurants. The restaurant nutrition environment is typically defined as the consumer nutrition environment, such as healthy options available within the restaurant, nutritional quality, prices, portion sizes, and promotions of the food served [13,14]. Traditional approaches to measuring the restaurant nutrition environment include business classification (e.g., full-service vs. limited-service restaurants) and store audits [15,16,17]. Among these measures, the Nutrition Environment Measures Survey in Restaurants (NEMS-R) emerges as a widely utilized store audit tool to evaluate the restaurant nutrition environment. The NEMS-R focuses primarily on the availability of nourishing main dishes, as well as the presence of fruits and vegetables, pricing, promotions, and other facilitators and barriers to healthy eating within restaurant settings [14]. However, these assessment measures, including the NEMS-R, do not account for individual dietary preferences and behaviors. Specifically, it is unknown to what extent the food items offered at the restaurant are actually purchased and consumed. To attain nutrition assessment on an individual level, a subset of studies has embraced more individualized approaches, employing dietary assessment tools such as biomarkers, 24-hour dietary recall (24HR), food frequency questionnaire (FFQ), and dietary record (DR) [18,19,20]. Nutritional biomarkers serve as clinical instruments for objectively gauging the presence of nutrients in biological samples, offering insights into the nutritional status of dietary intake or metabolic processes [19,21]. However, the data derived from biomarker measures can be biased by individual disease profiles, genetic background, homeostatic regulation, and the rigor of sample collection and storage procedures [22]. Other studies have opted to employ survey methods such as 24HR, FFQ, and DR to directly gather data on individual dietary intake [18]. However, these survey data are susceptible to potential recall bias and social desirability bias [18]. The data collection process in these methods may also lead to respondent burden, incur high costs, and involve time-intensive procedures, thereby restricting their feasibility for large-scale research.

Progress in food image capturing and recognition technologies offers alternative avenues for gathering and analyzing dietary intake data. The exploration of food image recognition was initially conducted within a university cafeteria setting. Digital photography was harnessed to capture facets such as food selections, food intake, and plate waste, subsequently undergoing comparison with the visual estimation method to ascertain its validity [23]. Later on, a study successfully achieved the remote and real-time collection of food intake data from individuals in their daily life contexts [24]. Participants were instructed to independently take photos of their food selections and plate waste, which they then transmitted to researchers [24]. A subsequent investigation employed a mobile phone application called Nutricam to document food intake [25]. This approach yielded a more comprehensive dietary dataset by combining participants’ captured food images with supplementary audio to further interpret the food content [25]. Propelled by the progress in computer and information technology, an increasing number of researchers have employed deep-learning models to automate the recognition of food images [26,27,28]. These deep learning-based food image recognition models can identify the food item and estimate its associated nutrition information [26,27,28,29,30]. When combined with a large volume of food image data that are readily available online, such as those collected through mobile apps or social media, this approach has the potential to offer a cost-effective and scalable solution to large-scale dietary or nutrition environment assessment [30]. Despite the potential, their integration into large-scale research endeavors remains largely underexplored.

To bridge this crucial gap, two exploratory studies used a deep learning-based food image recognition tool, called Calorie Mama, to estimate the nutrition information from food images [31,32]. These studies used crowdsourced food images from restaurants’ online presence (e.g., Google Place and Tripadvisor) and assessed the nutrition quality of the restaurant foods [31]. These studies also validated food image recognition as a viable and scalable tool for identifying and assessing restaurant foods. However, much remains unknown about the validity of the crowdsourced food images as the primary data source to evaluate the restaurant nutrition environment. It remains unknown to what extent crowdsourced food images represent the nutritional quality of the restaurant and the actual dietary choices of restaurant customers.

In this paper, using restaurants in the Greater Hartford region as a case study, we collect data from multiple sources and investigate the validity of using crowdsourced food images to evaluate the restaurant nutrition environment. Our analyses unfold across three distinct dimensions (i.e., participants, food items, and restaurants) and at multiple angles, including examining the representativeness of the social media platforms and those who post food images on social media, the consistency between information derived from crowdsourced food images and those derived from menus, residents’ perceptions, and GPS-based foot traffic data. To the best of our knowledge, this study is one of the first to offer a comprehensive examination of the validity of using crowdsourced food images to evaluate the restaurant nutrition environment. It can lay a foundation for future studies employing deep learning-based food image recognition methods and crowdsourced data.

## 2. Materials and Methods

### 2.1. Sample and Data

This study is an extension of a previous study in the same study area [31]. The study area includes Hartford and its surrounding towns (i.e., East Hartford, Glastonbury, Newington, South Windsor, West Hartford, Wethersfield, and Windsor). There were a total of 532 restaurants in this study area. After filtering out invalid, missing, and duplicated data, a final sample of 476 restaurants was identified from the dataset. Out of these 476 restaurants, 123 were categorized as full-service restaurants (as per the North American Industry Classification System [NAICS] code 722511), and 353 were classified as limited-service restaurants (NAICS code 722513). To validate the use of crowdsourced food image data for evaluating the restaurant nutrition environment from an overarching perspective, we collected data from multiple sources, including crowdsourced food image data, foot traffic data, menu items and their nutritional information data, and survey data. The crowdsourced food image data were collected in a previous study [31]. We utilized the “simple mass downloader” Chrome extension to conduct image collection from Google Place and Tripadvisor (posted by online users up to 2021), initially amassing 19,907 images. We chose these two online platforms as they were ranked among the top three business review platforms [33]. We manually filtered the image data, excluding images that met specific criteria: (1) staged or advertising-related images, (2) images featuring beverages, (3) images unrelated to food (e.g., buildings, dining scenes, and people), and (4) restaurants with fewer than five images. This final dataset comprised 15,908 food images from the restaurants in our sample. Each food image was recognized and nutritionally labeled by a deep learning-based food image recognition app (Calorie Mama [34]). Previous research confirms that Calorie Mama is highly accurate in recognizing food images for dietary assessment, boasting a top-1 accuracy of 63% and a top-5 accuracy of 88%. It also effectively identifies multiple components in mixed dishes [35].

The foot traffic data recorded the number of visits to each restaurant in our sample in 2018–2019 and were available for 359 restaurants (290 limited-service and 69 full-service) in the study area. This dataset was obtained from SafeGraph (Denver, CO, United States) Core Places and Patterns datasets, which were compiled from roughly 10% of mobile devices across the United States. It encompasses data regarding the number of visits from individuals’ residential census tracts to various points of interest (POIs). SafeGraph employs a verified algorithm to ascertain visits to POIs, with a requisite visit duration of a minimum of 4 min for it to be considered a visit to a specific POI [36,37]. Menu items were collected from Allmenus [38] in 2021, which covers a vast number of restaurants across the United States. In cases where information was unavailable on Allmenus, we obtained the menu items from the restaurants’ official websites. Around 100 restaurants’ menus were obtained from restaurants’ official websites, and in cases where the menu was in image format, manual transcription was performed. This yielded a total of 47,010 menu items. Each menu item was then matched to the USDA FoodData Central database [39] (i.e., an integrated data system that provides expanded nutrient profile data) and was nutritionally labeled. It is worth mentioning that approximately 4% of the menu items did not find a corresponding match in the database.

Finally, we conducted a Qualtrics survey to gather residents’ food image posting behavior on social media and perceptions of their favorite restaurants in the study area. In the survey, there were two sections relevant to this study. The first section included seven questions primarily focused on the frequency of posting food images on social media, preferred social media platforms for posting restaurant food images, details about participants’ most frequently visited restaurants (including name and location), and their perceptions of the nutrition quality of these restaurants. The second section comprised nine questions aimed at collecting sociodemographic data. The survey questions employed to evaluate residents’ perceptions of restaurant nutrition quality in the first section were adapted from the Perceived Nutrition Environment Measures Survey (NEMS-P), a validated tool for assessing perceived nutrition environments. The remaining questions were general inquiries related to food image posting behavior on social media and sociodemographic characteristics. The advertisement was primarily posted on Facebook and the survey was administered in July 2022. To be eligible for the survey, the participant had to be a resident of the study area, aged over 18 years, and willing to provide their home address. A total of 424 participants completed the survey.

### 2.2. Measures

Crowdsourced food images. For each restaurant, we recorded the number of food images posted online (log-transformed). The distribution of the number of food images was highly skewed, so we log-transformed them to be more normally distributed. We also estimated the calorie density of each food item (calories per 1 kg food, derived by Calorie Mama) and aggregated the information (taking the average) on the restaurant level.

Number of visits. We aggregated the SafeGraph data at the restaurant level and calculated the number of visits (log-transformed) to each restaurant in our sample in 2018–2019. Given that the distribution of the number of visits was highly skewed, we log-transformed the variable.

Calorie density from the restaurant menu. We collected the calorie density information of every menu item of each restaurant (calories per 1 kg food, collected from the FoodData Central database) and aggregated the information (taking the average) on the restaurant level.

Posting food images on social media. Participants’ preferences for social media to post restaurant food images were collected with a multiple-answer question: When you eat a meal away from home or get take-out food, which social media platform do you usually use to post food images? The options included: Facebook, Instagram, Twitter, Google, LinkedIn, Pinterest, Reddit, Snapchat, Discord, Tripadvisor, Tumblr, Yelp, and others. The total number of mentions of each social media platform in the responses was used to measure the popularity of social media platforms in terms of posting food images. Participants were also asked about the frequency of posting restaurant food images on social media using a Likert scale that comprised “always”, “very often”, “sometimes”, “rarely”, and “never”.

Restaurant perceptions. We asked survey participants to identify their most frequently visited restaurants in the study area and their favorite dish in the identified restaurant. To assess the residents’ perceptions of the restaurants’ nutrition quality, we adapted the 6-item questionnaire from the NEMS-P [40], which asked the participants to evaluate the restaurant’s availability of healthy options (2 survey items: there are many healthy menu options at the restaurant; it is easy to find healthy fruit and vegetable calories at the restaurant), the extent to which restaurants promote healthy options/nutrition information (3 survey items: the restaurant provides nutrition information on a menu board or the menu; signs and displays encourage overeating or choosing unhealthy foods from the menu; the menu or menu board highlights and promotes the healthy options at the restaurant), and the extent to which it costs more to buy healthy options (1 survey item: it costs more to buy healthy options). The NEMS-P survey items were presented to respondents in a matrix table, and they were asked to provide ratings on a 5-point Likert scale: (1) strongly disagree, (2) somewhat disagree, (3) neither agree nor disagree, (4) somewhat agree, and (5) strongly agree. Each response option was assigned a numerical value, where “strongly disagree” equated to 1 point, “somewhat disagree” to 2 points, “neither agree nor disagree” to 3 points, “somewhat agree” to 4 points, and “strongly agree” to 5 points. The averages of the participants’ scores for each construct of a restaurant were used as the NEMS-P scores of the restaurant for subsequent statistical analysis.

Survey participants’ sociodemographic characteristics. We also collected various sociodemographic characteristics of the survey participants, including gender, age, ethnicity, education, income, employment, marital status, and access to a car.

### 2.3. Analysis

To validate the use of crowdsourced food image data to evaluate the restaurant nutrition environment, we conducted multiple analyses on three levels. The first analysis was at the participant level, and the purpose was to evaluate (a) whether Google Place and Tripadvisor (where food images in this study were collected) were the appropriate platforms to collect restaurant food images, and (b) the extent to which those who posted restaurant food images online are representative of the population of local consumers. To that end, we assessed the participants’ preference for social media to post restaurant food images. Then, we summarized participants’ frequency of posting food images on social media by their sociodemographic characteristics, and a chi-squared test was performed to evaluate statistical significance.

The second analysis was at the food item level. The purpose was to evaluate the extent to which crowdsourced food images represent the food items that were available or actually ordered at the restaurant. To that end, we first matched the favorite restaurants that survey participants mentioned to the restaurants in our sample. For all the matched restaurants, we asked participants about their favorite dishes and calculated the proportion of the dishes that participants mentioned that also appeared in the crowdsourced food image data. Two independent coders also coded and matched the menu items from 120 randomly selected restaurants (60 full-service and 60 limit-service) with the crowdsourced food images, and we calculated the proportion of the menu items that appeared in the crowdsourced food image data for each restaurant.

**Figure 1:**
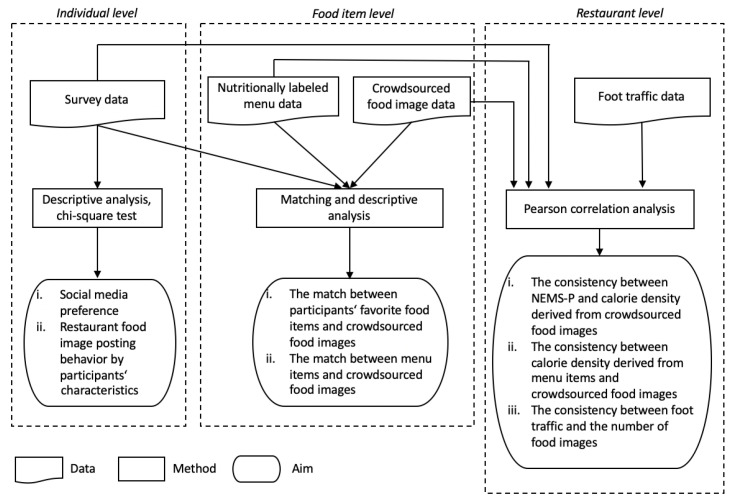
Overview of the study scheme at three levels: individual, food item, and restaurant. The dashed boxes represent different levels of analysis. The arrows indicate the data source(s) used for each analysis and/or the research objectives of each analysis.

The third analysis was at the restaurant level, and the purpose was to evaluate (a) the extent to which restaurant nutrition quality derived from crowdsourced food image data is consistent with that derived from survey participants’ perceptions or those from restaurant menus, and (b) the extent to which restaurant popularity derived from crowdsourced food image data is consistent with the actual foot traffic. To that end, we performed Pearson correlation analyses for three sets of variables at the restaurant level: (a) average calorie density derived from crowdsourced food images and each dimension of the NEMS-P scores from survey participants, (b) average calorie density derived from crowdsourced food images and that derived from the restaurant menu, and (c) the number of food images in the crowdsourced food image data and the number of visits from SafeGraph data. The distributions and summary statistics of all variables were examined prior to the analysis to ensure all statistical assumptions were met. All statistical analyses were performed using SPSS (version 28.0, SPSS Inc., Chicago, IL, USA) and SAS software (version 9.4, SAS Inc., Cary, NC, USA). Our study scheme is summarized in Figure 1.

## 3. Results

### 3.1. Participant Level

#### 3.1.1. Participant Characteristics and Social Media Preference

A total of 424 participants completed the survey. Table 1 presents the characteristics of the participants. The majority of the surveyed individuals identified themselves as White (70.3%), male (53.5%), aged between 25 and 34 (55.9%), and either married or having domestic partners (77.4%). Additionally, a significant portion possessed a 4-year college degree (28.8%), was employed for wages (71.2%), had an annual household income of 40,000–59,999 USD (34.9%), and had access to a car (90.8%).

**Figure 2:**
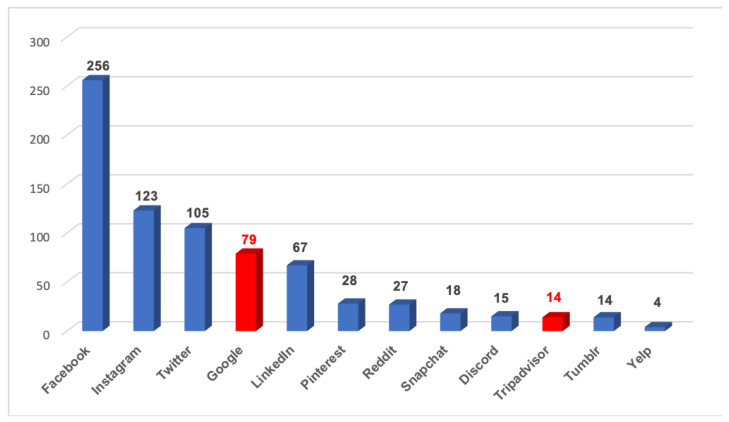
The preferred social media platforms to post restaurant food images. The red bars are the platforms used in the study; the blue bars are other platforms used by the participants.

Regarding their social media preferences, participants most commonly mentioned Facebook as the social media to post food images (38%), followed by Twitter (15%), Instagram (15%), Google (11%), LinkedIn (8%), and others, as shown in Figure 2.

#### 3.1.2. Restaurant Food Image Posting Behavior by Participants’ Characteristics

Table 2 illustrates participants’ frequency of posting restaurant food images on social media by their sociodemographic characteristics. It was observed that the frequency of such postings differed by factors including age, ethnicity, education, income, employment, marital status, and access to a car. Specifically, those in the age group of 25–34, with college experience, with an annual household income of 100,000–149,999 USD, and employed posted restaurant food images on social media much more frequently than other groups (all *p* < 0.0001). For example, 27% and 40% of the participants aged 25–34 answered “always” and “very often” when asked about their frequency of posting restaurant food images on social media, significantly higher than the rest of the participants (8% and 22% answered “always” and “very often”, respectively). Among those with a 4-year college degree, 49% answered “always”, while 44% of those with some college experience responded with “very often,” both significantly higher than the remaining participants (10% and 27% answered “always” and “very often” respectively). In the income bracket of 100,000–149,999 USD, 42% of participants reported posting “always”, considerably higher than the other participants (14%), and 22% of employed participants answered “always,” which was significantly higher than the other participants (11%).

### 3.2. Food Item Level

#### 3.2.1. Participants’ Favorite Food Items and Crowdsourced Food Images

We matched the restaurants that survey participants mentioned to the restaurants in our sample. Of the 423 participants, 222 individuals (46%) had their most frequently visited restaurants matched with the restaurant list in our study sample (83 unique restaurants). Among these 222 participants, 147 favorite food items they mentioned were valid and 70.1% of the favorite foods they mentioned had a match in the crowdsourced food image dataset.

#### 3.2.2. Menu Items and Crowdsourced Food Images

Two independent raters coded and matched restaurant menu items with crowdsourced food images using a random sample of 120 restaurants (60 full-service and 60 limited-service), and calculated the proportion of the menu items that appeared in the crowdsourced food image dataset. The distribution of the matched percentages for all restaurants and different types of establishments (full-service vs. limited-service) is presented in Table 3. Overall, the mean match rate was 44% (standard deviation [SD] = 18%, ranging from 2% to 96%). Full-service restaurants had an average match rate of 40% (SD = 12%, ranging from 2% to 67%), while limited-service restaurants had a slightly higher mean match rate of 48%, but a considerably large variation (SD = 22%, ranging from 7% to 96%).

### 3.3. Restaurant Level

#### 3.3.1. NEMS-P Scores and Average Calorie Density

Table 4 illustrates the correlation between average calorie density (derived from the crowdsourced food images) and different dimensions of the NEMS-P score at the restaurant level (n = 83). Participants’ perceptions that healthy options are more costly at the restaurant had a weak positive correlation with the average calorie density of the restaurant (r = 0.24, *p* = 0.03). No significant correlations were found for the other two dimensions of NEMS-P scores.

#### 3.3.2. Calories Derived from Menu Items and Food Image Recognition

Table 5 presents the correlation between average calorie density derived from food images and those obtained from menu items. A total of 419 restaurants were included in this analysis after excluding those with missing values in either dataset. Overall, there was only a weak positive correlation (r = 0.16, *p* = 0.001) between the two and the relationship did not differ much by restaurant type.

#### 3.3.3. Foot Traffic and Number of Food Images

Pearson correlation analysis revealed that there was only a weak positive correlation between the number of visits/foot traffic and the number of posted food images (both variables were log-transformed) across restaurants (r = 0.14, *p* = 0.007). The correlation was stronger for full-service restaurants (r = 0.24, *p* = 0.047) than for limited-service restaurants (r = 0.11, *p* = 0.063) (Table 6).

## 4. Discussion

This paper is one of the first studies assessing the validity of utilizing crowdsourced food image data to evaluate the restaurant nutrition environment. Analyses were conducted at three distinct levels—participant, food item, and restaurant—focusing especially on all restaurants within the Greater Hartford region, Connecticut. The results show that crowdsourced image data from social media platforms hold promise as a supplementary and cost-effective means for assessing the restaurant nutrition environment. However, they should be employed with caution, given their partial validity.

Our findings reveal a consistent pattern in the characteristics of participants who posted restaurant food images on social media. Specifically, our finding shows gender is not significant in predicting the frequency of posting behavior. While this finding is in line with some studies [41,42], other studies found that there is a gender difference in posting images on social media [43,44]. We found that there is a higher frequency of posting behavior from employed younger individuals with college experience and an annual household income ranging from 100,000 to 149,999 USD compared to other groups. This result suggests that social media users who post restaurant food images cannot represent the general population of restaurant customers, as there is an overrepresentation of young people with higher education attainments. This observation can be attributed to the presence of a digital divide in the utilization of social media [45]. Neighborhoods with different demographic characteristics may exhibit significant disparities in internet and mobile device access, as well as social media usage, impacting the coverage and representativeness of restaurant food images in specific areas [46]. Given these insights, analysis of online food image data must be supplemented with other forms of data (e.g., surveys and menu labeling data) to reliably assess the restaurant nutrition environment.

Our results show that Facebook, Twitter, and Instagram were the most prevalent social media platforms for posting food images, while Google and Tripadvisor were less frequently used by participants. It is worth mentioning that this outcome may be somewhat biased, given that the survey’s primary recruitment was through Facebook. This finding suggests that using Google and Tripadvisor as the primary platforms for capturing restaurant consumers’ dietary behaviors requires further assessment. These two platforms were utilized in this study because they provide a comprehensive list of restaurants for users to review, whereas other social media platforms had limited restaurant listings. To leverage the advantages of different types of social media platforms, future studies could explore the use of alternative platforms (e.g., Facebook and Twitter) where restaurants maintain an online presence [47,48,49]. Previous studies have demonstrated the effectiveness of utilizing Twitter data to perform nutrition assessments in specific communities and populations. For instance, Chen et al. [50] collected individual data from Twitter to explore the link between the food environment and the quality of food choices. Vydiswaran et al. [51] examined the validity of Twitter review data to characterize neighborhood-level food-related behaviors and attitudes. Additionally, Nguyen et al. [52] employed Twitter data to establish food environment indicators relevant to public health intervention. By utilizing diverse social media platforms, researchers can obtain a more comprehensive and inclusive understanding of the community nutrition environment and food-related consumer behaviors.

Our findings at the food item level show that 70.1% of the favorite restaurant foods that participants mentioned had matches in the food image dataset. This result indicates that while crowdsourced food image data overrepresent the dietary behaviors of certain restaurant customers, they might still be a reliable and useful tool to identify popular items in the restaurant. Overall, only 44% of the menu items appeared in the crowdsourced food image data. The low match rate is somewhat expected, as not all menu items are frequently ordered by customers [53] and the selection frequency can sometimes be influenced by visual stimuli, the positioning of menu items, and the order in which they are presented [54,55]. More specifically, full-service restaurants had a slightly lower match rate (40%) compared to limited-service restaurants (48%), which could be partly due to the longer and more complicated menus at the full-service restaurants [56].

At the restaurant level, the average calorie density derived from the crowdsourced food images shows (a) weak positive correlations with participants’ perceptions that healthy options are more costly at the restaurant and no correlations with other dimensions of NEMS-P, and (b) weak positive correlations with the average calorie density obtained from menu items. The latter result is expected, given the low match rate between menu items and the food image data, while the former indicates that restaurant nutrition quality derived from food images is largely inconsistent with consumers’ perceptions, possibly due to the aforementioned reasons (e.g., online food images overrepresent certain types of restaurant customers and popular food items). This result aligns with a previous study that combined crowdsourced Yelp data and a Nutrition Environment Measures Survey for Stores (NEMS-S) to evaluate the consumer nutrition environment in grocery stores, which unveiled that there is no significant correlation between NEMS-S scores on food availability, quality, and price and the sentiment extracted from the social media data [46]. Similarly, there was only a weak positive correlation between the number of images and foot traffic across restaurants, and the correlation was stronger for full-service restaurants (r = 0.24) compared to limited-service restaurants (r = 0.11). This result indicates that the popularity of restaurants gauged through the online survey does not represent the actual observed foot traffic, especially for limited-service restaurants. Customers may patronize limited-service restaurants more frequently without posting any food images on social media, as people derive more pleasure from their dining experiences and those with hedonistic inclinations tend to post food images on social media [57,58].

This study also has limitations. First, we acknowledge several inherent limitations stemming from the various data sources employed. In our analysis of crowdsourced food image data, we focused only on calorie density. Future research should consider exploring additional macronutrient and micronutrient information for restaurant food and assess overall health implications using comprehensive indices such as the Healthy Eating Index. Regarding the foot traffic data acquired from SafeGraph, it is essential to note that takeaway visits lasting less than 4 min were excluded from our analysis, and the same for delivery services. The nutrition data extracted from the FoodData Central database provided only a general nutrition profile and may not fully reflect each restaurant’s specific food preparation methods. Furthermore, our survey questions only asked generally about food image posting behavior without asking about the purpose of posting. Future studies may further expand survey questions and distinguish between different purposes of restaurant food image posting, such as sharing with family and friends or creating formal restaurant reviews. Second, a temporal mismatch exists among the multiple datasets used, as survey data, crowdsourced image data, and menu items were collected at different time points over a 4-year period, which could affect the correlations among some measures. Third, the survey results might be biased to some degree, as the majority of survey participants were recruited from Facebook and were employed young individuals with college experience, which might not fully represent the diverse populations in the study area. Finally, the small sample of the matched restaurants (n = 83) and matched favorite food items (n = 147) from the survey and the study’s regional focus may limit the generalizability of the findings.

## 5. Conclusions

Leveraging crowdsourced food image data for assessing restaurant nutrition environments holds much potential due to its cost-effectiveness and scalability. In this study, we have used data from multiple sources to investigate the validity of this approach. Our results indicate that crowdsourced food image data can be useful in the initial assessment of restaurant nutrition quality and the identification of popular food items. However, they are inherently susceptible to selection bias on multiple levels and do not fully represent the restaurant’s nutrition quality or the perception and dietary behaviors of restaurant customers. If employed, the food image data must be supplemented with alternative data sources, such as field surveys, store audits, and commercial data, to offer a more representative assessment of the restaurant nutrition environment.

## Figures and Tables

**Table 1:** Survey participant characteristics (N = 424).

Characteristics	N (%)
Gender	
Male	227 (53.5%)
Female	195 (46%)
Other	2 (0.2%)
Age	
18–24	18 (4.2%)
25–34	237 (55.9%)
35–44	136 (32.1%)
45–54	20 (4.7%)
55–64	6 (1.4%)
65 and above	7 (1.7%)
Race	
American Indian or Alaska Native	21 (5%)
Asian	32 (7.5%)
Black or African-American	64 (15.1%)
Native Hawaiian or Other Pacific Islander	8 (1.9%)
White	298 (70.3%)
Other	3 (0.7%)
Education	
Finished middle school	12 (2.8%)
Finished high school or got a GED	51 (12%)
Some college	117 (27.6%)
Completed a 2-year college degree	94 (22.2%)
Completed a 4-year college degree	122 (28.8%)
Completed a graduate degree	27 (6.4%)
Income (USD)	
Less than 20,000	8 (1.9%)
20,000–39,999	51 (12%)
40,000–59,999	148 (34.9%)
60,000–79,999	78 (18.4%)
80,000–99,999	56 (13.2%)
100,000–149,999	66 (15.6%)
150,000–199,999	10 (2.4%)
200,000 and above	7 (1.7%)
Employment	
A homemaker	8 (1.9%)
A student	10 (2.4%)
Employed for wages	302 (71.2%)
Military	3 (0.7%)
Out of work and looking for work	32 (7.5%)
Out of work but not currently looking for work	22 (5.2%)
Self-employed	42 (9.9%)
Retired	5 (1.2%)
Marital status	
Married or domestic partnership	328 (77.4%)
Single, never married	78 (18.4%)
Widowed, divorced, or separated	18 (4.2%)
Access to a car	
Yes	385 (90.8%)
No	39 (9.2%)

**Table 2:** Characteristics of the survey participants and the frequency of posting food images on social media.

	Always	Very Often	Sometimes	Rarely	Never	Total
Gender (chi-squared = 7.5, *p* = 0.5)						
Male	38 (17%)	78 (34%)	71 (31%)	19 (8%)	21 (9%)	227
Female	39 (20%)	58 (30%)	50 (26%)	24 (12%)	24 (12%)	195
Other	1 (50%)	0 (0%)	1 (50%)	0 (0%)	0 (0%)	2
Age (chi-squared = 108.2, *p* < 0.0001)						
18–24	1 (6%)	5 (28%)	7 (39%)	4 (22%)	1 (6%)	18
25–34	63 (27%)	94 (40%)	51 (22%)	16 (7%)	13 (5%)	237
35–44	12 (9%)	33 (24%)	54 (40%)	18 (13%)	19 (14%)	136
45–54	2 (10%)	3 (15%)	10 (50%)	1 (5%)	4 (20%)	20
55–64	0 (0%)	0 (0%)	0 (0%)	3 (50%)	3 (50%)	6
65 and above	0 (0%)	1 (14%)	0 (0%)	1 (14%)	5 (71%)	7
Race (chi-squared = 35.1, *p* = 0.02)						
American Indian or Alaska Native	1 (5%)	3 (14%)	8 (38%)	4 (19%)	5 (24%)	21
Asian	4 (13%)	5 (16%)	12 (38%)	3 (9%)	8 (25%)	32
Black or African-American	11 (17%)	22 (34%)	19 (30%)	9 (14%)	3 (5%)	64
Native Hawaiian or Other Pacific Islander	0 (0%)	2 (25%)	4 (50%)	1 (13%)	1 (13%)	8
White	62 (21%)	104 (35%)	78 (26%)	25 (8%)	27 (9%)	296
Other	0 (0%)	0 (0%)	1 (33%)	1 (33%)	1 (33%)	3
Education (chi-squared = 90.7, *p* < 0.0001)						
Finished middle school	3 (25%)	2 (17%)	5 (42%)	2 (17%)	1 (8%)	12
Finished high school or got a General Educational Development (GED)	5 (10%)	17 (33%)	21 (41%)	3 (6%)	5 (10%)	51
Some college	10 (9%)	52 (44%)	31 (26%)	10 (9%)	14 (12%)	117
Completed a 2-year college degree	4 (4%)	31 (33%)	40 (43%)	12 (13%)	7 (7%)	94
Completed a 4-year college degree	49 (40%)	31 (25%)	18 (15%)	13 (11%)	11 (9%)	122
Completed a graduate degree	7 (26%)	3 (11%)	7 (26%)	3 (11%)	7 (26%)	27
Income (USD) (chi-squared = 123.0, *p* < 0.0001)						
Less than 20,000	0 (0%)	2 (25%)	1 (13%)	5 (63%)	0 (0%)	8
20,000–39,999	6 (12%)	17 (33%)	21 (41%)	3 (6%)	4 (8%)	51
40,000–59,999	30 (20%)	62 (42%)	34 (23%)	13 (9%)	9 (6%)	148
60,000–79,999	7 (9%)	28 (36%)	28 (36%)	8 (10%)	7 (9%)	78
80,000–99,999	5 (9%)	7 (13%)	25 (45%)	7 (13%)	12 (21%)	56
100,000–149,999	28 (42%)	17 (26%)	10 (15%)	4 (6%)	7 (11%)	66
150,000–199,999	2 (20%)	2 (20%)	3 (30%)	2 (20%)	1 (10%)	10
200,000 and above	0 (0%)	1 (14%)	0 (0%)	1 (14%)	5 (71%)	7
Employment (chi-squared = 78.5, *p* < 0.0001)						
A homemaker	1 (13%)	3 (38%)	3 (38%)	0 (0%)	1 (13%)	8
A student	0 (0%)	3 (30%)	2 (20%)	4 (40%)	1 (10%)	10
Employed for wages	65 (22%)	102 (34%)	77 (25%)	24 (8%)	34 (11%)	302
Military	0 (0%)	1 (33%)	1 (33%)	1 (33%)	0 (0%)	3
Out of work and looking for work	4 (13%)	11 (34%)	11 (34%)	5 (16%)	1 (3%)	32
Out of work, but not currently looking for work	2 (9%)	4 (18%)	10 (45%)	5 (23%)	1 (5%)	22
Self-employed	6 (14%)	12 (29%)	18 (43%)	4 (10%)	2 (5%)	42
Retired	0 (0%)	0 (0%)	0 (0%)	0 (0%)	5 (100%)	5
Marital status (chi-squared = 20.4, *p* = 0.01)						
Married or domestic partnership	68 (21%)	110 (34%)	91 (28%)	24 (7%)	35 (11%)	328
Single, never married	9 (12%)	23 (29%)	23 (29%)	16 (21%)	7 (9%)	78
Widowed, divorced, or separated	1 (6%)	3 (17%)	8 (44%)	3 (17%)	3 (17%)	18
Access to a car (chi-squared = 18.4, *p* = 0.001)						
Yes	78 (20%)	124 (32%)	110 (29%)	33 (9%)	40 (10%)	385
No	0 (0%)	12 (31%)	12 (31%)	10 (26%)	5 (13%)	39

**Table 3:** Distribution of the match rate between restaurant menu items and food image datasets.

	Mean (SD)	Range
All restaurants (n = 120)	44% (18%)	2–96%
Full-service (n = 60)	40% (12%)	2–67%
Limited-service (n = 60)	48% (22%)	7–96%

**Table 4:** Correlations between average calorie density (estimated by the image recognition model) and each NEMS-P composite item score of the restaurants.

Composite Item		Availability of Healthy Options	Restaurant Promotes Healthy Options/Nutrition Information	Costs More to Buy Healthy Options
Model estimated calorie density of the restaurant	Correlation coefficient ^®^	0.06	0.17	0.24
	*p*	0.6	0.1	0.03
	Cronbach’s alpha (α)	0.5	0.5	na

na not applicable–Cronbach’s alpha cannot be calculated with only one item.

**Table 5:** Correlations between average calorie density estimated by the image recognition model and average calorie density derived from menu items.

		Calories Calculated from the Menu: All Restaurants (n = 419)	Calories Calculated from the Menu: Full-Service Restaurants (n = 114)	Calories Calculated from the Menu: Limited-Service Restaurants (n = 305)
Calories estimated by the image recognition model	Pearson Correlation	0.16 **	0.17	0.17 **
	*p*	0.001	0.076	0.002

** *p* < 0.01.

**Table 6:** Correlations between foot traffic and the number of food images for the restaurants in the study.

		Number of Images of Total Restaurants from Food Image Dataset (n = 359)	Number of Images of Full-Service Restaurants from Food Image Dataset (n = 69)	Number of Images of Limited-Service Restaurants from Food Image Dataset (n = 290)
Foot traffic from SafeGraph	Pearson Correlation	0.14 **	0.24 *	0.11
	*p*	0.007	0.047	0.063

* *p* < 0.05, ** *p* < 0.01.

## Data Availability

The data presented in this study are available upon request from the corresponding author. The data are not publicly available due to privacy restrictions.
